# Role of TLR4/NF-κB in Damage to Intestinal Mucosa Barrier Function and Bacterial Translocation in Rats Exposed to Hypoxia

**DOI:** 10.1371/journal.pone.0046291

**Published:** 2012-10-17

**Authors:** Han Luo, Ping Guo, Qiquan Zhou

**Affiliations:** 1 Department of High Altitude Diseases, College of High Altitude Military Medicine, Third Military Medical University, Chongqing, China; 2 Key Laboratory of High Altitude Medicine of Ministry of Education and Key Laboratory of High Altitude Medicine of PLA, Chongqing, China; Charité, Campus Benjamin Franklin, Germany

## Abstract

The role of Toll-like receptor 4 (TLR4)/nuclear factor-kappa-B (NF-κB) in intestinal mucosal barrier damage and bacterial translocation under hypoxic exposure is unclear. Here, we investigated their role using an acute hypobaric hypoxia model. Adult Sprague-Dawley rats were divided into control (C), hypoxia (H), hypoxia+NF-κB inhibitor pyrrolidinedithiocarbamic acid (PDTC) (100 mg. kg) (HP), hypoxia+0.5 mg/kg lipopolysaccharide (HPL), and hypoxia+PDTC+LPS (HPL) group. Except control group, other four groups were placed in a hypobaric chamber set at 7000 m. Samples were collected at 72 h after pressure reduction. Damage in ultrastructure of the intestinal tract was examined by transmission electron microscopy and bacterial translocation was detected by cultivation. Kinetic turbidimetric assay was used to measure the serum LPS.

ELISA was performed to detect TNF-α and IL-6 serum concentrations. Fluorescent quantitative RT-PCR was used to measure TLR4 mRNA levels was measured using quantitative RT-PCR and protein of NF-κB p65 was measured by western blotting. Different degrees of intestinal mucosa damage were observed in groups H and HL. The damage was significantly alleviated after blockage of the TLR4/NF-κB signaling pathway. PDTC- treatment also reversed hyoxia- and LPS-induced bacterial translocation rate and increased serum levels of LPS, TNF-α, and IL-6. TLR4 mRNA levels and NF-κB p65 expression were consistent with the serum factor results. This study suggested that TLR4 and NF-κB expression increased in rat intestinal tissues after acute hypoxia exposure. PDTC-treatment reversed TLR4 and NF-κB upregulation and alleviated damage to the intestinal tract and bacterial translocation. Thus, the TLR4/NF-κB signaling pathway may be critical to the mechanism underlying hypoxia-induced damage to intestinal barrier function and bacterial translocation.

## Introduction

High altitudes create a special type of environment, incorporating both low pressure and low oxygen levels. High altitude hypoxia causes severe damage to different organs, especially the intestinal tract. The incidence of digestive system disease is reported to be quite high among high-altitude residents and immigrants [1], [2]. The intestinal tract is the largest reservoir of bacteria and endotoxins in the human body. Once intestinal bacteria and endotoxins enter the portal vein or lymphatic system, they can translocate to other tissues and organs [3], leading to a cascade response mediated by inflammatory mediators [3], including TNF-α and IL-6. This can induce systemic inflammatory response syndrome or even multiple organ dysfunction syndrome (MODS) [4], [5], which further damage the function of the intestinal barrier. During plateau gastrointestinal tract damage, impairment of the function of the intestinal barrier and intestinal bacterial translocation are the starting points of many acute high-altitude illnesses. Currently, however, their underlying mechanisms are still unknown.

Toll-like receptors (TLRs) are a family of pattern-recognition receptors that play a key role in the innate immune system. Subtype TLR4 is expressed at high levels in the intestinal tract, and its specific ligand lipopolysaccharide (LPS) is widely distributed among intestinal bacteria. For this reason, TLR4 is thought to be involved in the first immune barrier of the gastrointestinal tract that recognizes bacteria. Nuclear factor-κB (NF-κB) is the final effector molecule of the TLR4 signaling pathway. It promotes the development of many intestinal diseases, but also inhibits inflammation. It also plays a pivotal role in the translation and transcription of inflammatory mediators [6]. Recent studies have shown that hypoxia alone can damage the function of the gastrointestinal barrier and cause flora imbalance in rats [7]. As a key transmembrane protein closely related to bacterial recognition, TLR4 transfers extracellular antigen information into cells and induces inflammatory reactions. It provides a new research target for studies of bacterial translocation and damage to the function of the intestinal barrier under hypoxic conditions.

The paracellular and transcellular pathways are two major pathways mediating transmembrane transfer of intestinal bacterial and substance. Both pathways might be involved in hypoxia-induced intestinal mucosal barrier damage and bacterial translocation. Paracellular pathway has an important component named tight junction (TJ) that consists of occludin, claudins, and cytoskeleton proteins. Previous studies have shown that hypoxia, inflammatory cytokines, and bacterial antigen can affect expression level and assembly of claudins, therefore exerted an influence on TJ functions. While occludin plays a critical role in TJ functions, the report about the role of occludin in the intestinal barrier has been very limited.

In the present study, we used hypoxia to generate an animal model with intestinal bacterial translocation and barrier damage. To investigate the role TLR4/NF-κB in the functional damage in this model, we evaluate changes in the expression of TLR4, NF-κB, and related inflammatory mediators such as tumor necrosis factor-alpha (TNF-α) and interleukin 6 (IL-6) after the application of the NF-κB inhibitor PDTC. In addition, we investigated the involvement of paracelluar pathway in the intestinal damage by evaluating the expression of this critical protein occludin.

## Materials and Methods

### Experimental animals

One hundred adult male Sprague-Dawley (SD) rats (200±20 g) were purchased from the Research Animal Center of the Research Institute of Surgery, Third Military Medical University. All procedures were approved by the Experimental Animals Committee of the Third Military Medical University. The disposal of experimental animals is strict comply with the management requirements of experimental animals.

### Reagents

The main agents used in this study were lipopolysaccharides (LPS) and NF-κB inhibitor PDTC (Sigma, USA); MacConkey agar plates (Pang Tong Medical Devices Co. Ltd., China); KTA TAL reagent (A&C Biological Ltd., China); rat IL-6 ELISA kit (Boster Bio-engineering Co. Ltd., China); rat TNF-α ELISA kit (Dakewe Biotech Co. Ltd., China); RNAiso Plus, PrimeScript reverse transcription kit, and SYBR Premix Ex Taq fluorescent quantitative PCR kit (TaKaRa, China); RQ1 RNase-Free DNase (Promega, USA); NF-κB p65 rabbit polyclonal antibody and β-actin mouse polyclonal antibody (Santa Cruz, USA); and occludin rabbit polyclonal antibody (Invitrogen, USA).

### Establishment of the animal model

One hundred rats were randomly divided into 5 groups of 20 rats each: control (group C), hypoxia alone (group H), hypoxia+PDTC (group HP), hypoxia+LPS (group HL), and hypoxia+PDTC+LPS (group HPL). The animals in group C were placed in a normal environment, while those in groups H, HP, HL, and HPL were placed in a hypobaric chamber simulating an elevation of 7000 m for 3 days. The rats in groups HP and HPL were given 100 mg/kg PDTC via an abdominal injection at 1 h prior to reducing the pressure. The rats were injected once a day for 3 consecutive days. The rats in groups HL and HPL were given 0.5 mg/kg LPS via an abdominal injection at 48 h after reducing the pressure. The groups that did not receive drugs received abdominal injections of saline of the same volume at the same time.

### Animal observation and sample collection

Samples were collected from all groups after 72 h. Animals were subjected to intraperitoneal anesthesia by using 10% urethane (1 g/kg). For group C, the samples were collected in the normal environment; for the other 4 groups, the animals were placed at a simulated altitude of 4500 m. The samples were collected quickly.

General conditions of the rats and overall changes in the abdominal cavity were observed. The rats were monitored for alertness and activity and for expansion, congestion, and edema of the intestinal canal in the abdominal cavity or any other notable instances of inflammation.

Further, bacterial translocation in the intestinal tract was assessed. Under sterile conditions, the thoracic and abdominal cavities were opened, and 1 mL blood was collected from the heart. One hundred milligram samples of mesenteric lymph node, liver, and spleen were separately homogenized in 900 µL sterile saline. Next, 0.5 mL whole blood and 0.5 mL of tissue homogenates from different organs were spread on MacConkey solid media and cultured at 37°C for 24 h before being subjected to bacterial colony counting and preliminary biochemical identification. Abdominal cavity swabs were also collected and incubated in LB liquid medium at 37°C for 24 h. These samples were then seeded on MacConkey solid media using the method described above. This served as the negative control. The rate of bacterial translocation was determined using the following equation: rate of bacterial translocation (%) = number of positive organs in the same bacterial culture/total number of organs examined.

### Measurement of serum endotoxin levels

Akinetic turbidimetric assay was used (following the instructions of the TAL test kit) to measure the serum endotoxin levels. For each group, 3 mL blood was collected from the apex of the heart, placed in a pyrogen-inactivated coagulation-promoting tube, and centrifuged at 3000 rpm for 10 min at 4°C to collect 500 µL serum. Then, 100 µL of serum was added to 900 µL KTA TAL reagent, mixed, and allowed to settle at 65°C for 20 min. We added 200 µL of this mixture to the main reagents of the enzymatic reaction. The samples were inserted into the EDS-99 Bacterial Endotoxin Dynamic Testing System for 60 min and the endotoxin level in each sample was detected automatically by the software.

### Transmission electron microscopy (TEM)

Lanthanum nitrate tracer was prepared for the observation of the ultrastructure of the intestinal tract using transmission electron microscopy. Each animal was anesthetized using 10% urethane (1 g/kg). The left external jugular vein was isolated, intubated, and 0.5 mL 2% lanthanum nitrate in saline was slowly injected. After 10 min, the chest was opened quickly and the aorta was intubated through the left ventricle; 0.9% physiological saline at 37°C was first used for fast perfusion (pressure maintained at 100 mmHg, approximately 200 mL total volume), and the right auricle was cut open until the fluid flowing out was mostly clear. Next, lanthanum aldehyde fixative solution (2% paraformaldehyde, 2.5% glutaraldehyde, 2% lanthanum nitrate, and 0.1 M sodium cacodylate) was used for perfusion under constant pressure for 30 min [8], [9]. A 2 cm long section of jejunum, located at 10 cm from the pylorus, was removed and then cut along the vertical axis to make 1 mm×1 mm×1 mm samples, which were immersed in lanthanum aldehyde fixative solution at 4°C for 24 h. The samples were rinsed 3 times for 30 min each with 0.1 M sodium cacodylate buffer (pH 7.4) at 4°C, and fixed with 1% osmic acid before another round of rinsing with sodium cacodylate buffer, as described above. The samples were subjected to gradient dehydration using acetone, embedded in epoxy resin 618, adjusted for shape, and block-localized using semi-thin sectioning. Ultra-thin slices were prepared and doubled stained with uranium-lead. TEM (TECNAI10, Philips) was used to visualize the ultrastructure of the intestinal tract and the distribution of lanthanum nitrate particles.

### RT-PCR for detection of TLR4 mRNA levels in the intestinal tract

Sixty milligrams (mg) of accurately weighed jejunal tissues was used for total RNA extraction using RNAiso Plus. Nanodrop ultramicro-ultraviolet spectrophotometry was used to measure concentration and purity. The criteria for successful extraction were an A260/A280 ratio between 1.9 and 2.1 and a concentration above 100 ng/µL. From each group, 500 ng of total RNA was extracted and subjected to residual genomic DNA inactivation using RQ1 RNase-Free DNase according to the manufacturer's instructions. The final concentration of total RNA was approximately 150 ng/µL for each group.

A PrimeScript® RT Master Mix Perfect Real Time Kit was used for reverse transcription. The total volume was 10 µL, including 2 µL of 5× PrimeScript® RT Master Mix, 1 µL total RNA, and 7 µL RNase-free dH_2_O. The reaction conditions were as follows: 37°C for 15 min and 85°C for 5 s.

The primers for fluorescent quantitative PCR were synthesized by Invitrogen Inc., and no homologous sequences were found after an NCBI BLAST search. The TLR4 primers were as follows: up primer, 5′-ATCTCAGCAAAATCCCTCAT-3′; down primer, 5′-AATCCAGCCACTGAAGTTGT -3′; the product length was 123 bp. The primers for the internal control gene (β-actin) were as follows: up primer, 5′-GTGGGCCGCC CTAGGCACCA-3′; down primer, 5′-CGGTTGGCCTTAGGGTTCAGAGGG-3′; the product length was 250 bp. A SYBR Premix Ex Taq™ II kit was used. The total volume was 20 µL, including 10 µL SYBR Premix Ex Taq™ II, 1.6 µL up and down primers (0.8 µL each), 1.6 µL cDNA template, and 6.8 µL dH_2_O. An MT Opticon Real-Time Fluorescent Quantitative PCR System was used for PCR with the following parameters: pre-denaturation at 95°C for 30 s; 40 cycles of 95°C for 5 s, 54°C for 30 s, and 72°C for 15 s; and 72°C for 5 min. Amplification plots and dissociation curves were constructed. On the basis of the Ct values of the target gene and of the internal control gene, which were collected automatically by the system, the 2^−ΔΔC^_t_ method was used to calculate the relative expression levels of TLR4 in each group.

### Western blotting for detection of NF-κB p65 and occludin expression

Eighty mg jejunal tissue was collected from each group, added to 800 µL RIPA lysate and 8 µL PMSF, and homogenized by ultrasound. Then, total protein was extracted following the manufacturer's instructions. The BCA method was used to measure protein concentration, and all protein solutions were diluted to 3 µg/µL with distilled water before addition to 5× SDS loading buffer. The mixtures were denatured for 5 min at 100°C. Proteins were loaded at 35 µg/µL, and a 5% spacer gel and 10% separation gel were used for electrophoresis at 80 V for 90 min. The protein bands were subjected to wet transfer at 100 V for 1 h to a PVDF membrane. A 5% fat-free milk solution was used for blocking at room temperature for 1 h. NF-κB p65 (1∶500), occludin (1∶500), and β-actin (1∶1000) primary antibodies were added, incubated at 4°C overnight, and rinsed 3 times with TBST for 10 min each before the addition of the HRP-labeled secondary antibody (1∶3000) at room temperature for 1 h. The membranes were rinsed 3 times with TBST for 10 min each. An ECL agent was added for chemiluminescence imaging. The images were collected using the Gel EQ System (Bio-Rad, Inc.), and the built-in software was used to analyze the gray values of the bands. The relative expression levels of the proteins were expressed as the gray value of the target band over the gray value of β-actin in the same sample. Each sample had 3 replicates.

### ELISA for detection of serum levels of TNF-α and IL-6

Rat serum was collected as described previously. ELISA was used to detect the serum levels of TNF-α and IL-6 in strict accordance with the manufacturer's instructions. A Synergy HT Microplate Reader was used to read the optical density at 450 nm, and the concentration of the sample was determined using a standard curve.

### Statistical analysis

SPSS 18.0 software was used for statistical analysis. Qualitative data are expressed in percentages, and quantitative data are expressed as mean ± standard deviation. One-way ANOVA was used for comparisons between groups; the t-test was used to compare the mean values of the samples from different groups; and the χ^2^ test was used to compare rates of bacterial translocation. Differences were considered statistically significant at *P*<0.05.

## Results

### General condition of the rats

The rats in group C (control) were active, energetic, and lacked any inflammatory reactions in the abdominal cavity that were visible to the naked eye. The rats in groups H and HL were less active and were notably more tired. Prominent swelling was observed in their intestinal canals, and the intestinal mucosa was congested. A small amount of ascites and mesenteric lymph node swelling was observed in the rats from group HL. The rats in groups HP and HPL also showed reduced activity and appeared relatively tired. Mild swelling was observed in their intestinal cavities, but there was no notable mucosa congestion.

### Bacterial translocation in the intestinal tract

Bacterial cultures of different organs collected from the rats in group C were all negative. The rates of bacterial translocation in groups H and HL were significantly different from those of group C (*P*<0.01), suggesting that hypoxia and hypoxia with LPS induced significant bacterial translocation in the intestinal tract. Moreover, the rate in HP and HPL group was significantly reduced compared to H and HL group (p<0.05, **Table 1**), respectively. This result suggested that NF-κB inhibitor PDTC reversed hypoxia and LPS-induced bacterial translocation in the intestinal tract.

**Table 1:** Rate of bacterial translocation in rats from different groups.

Group	n	Blood	Liver	Spleen	Lymph node	Translocation rate (%)
C	20	0	0	0	0	0
H	20	0	1	3	3	35^a^ ^c^
HP	20	0	0	1	0	5^b^
HL	20	0	4	5	5	70^a^
HPL	20	0	0	2	4	30^c^

Note:

a *P*<0.01 compared to group C;

b *P*<0.01 compared to group H;

c *P*<0.01 compared to group HL.

### Changes of the serum levels of LPS and the inflammatory mediators TNF-α and IL-6

**Figure 1:**
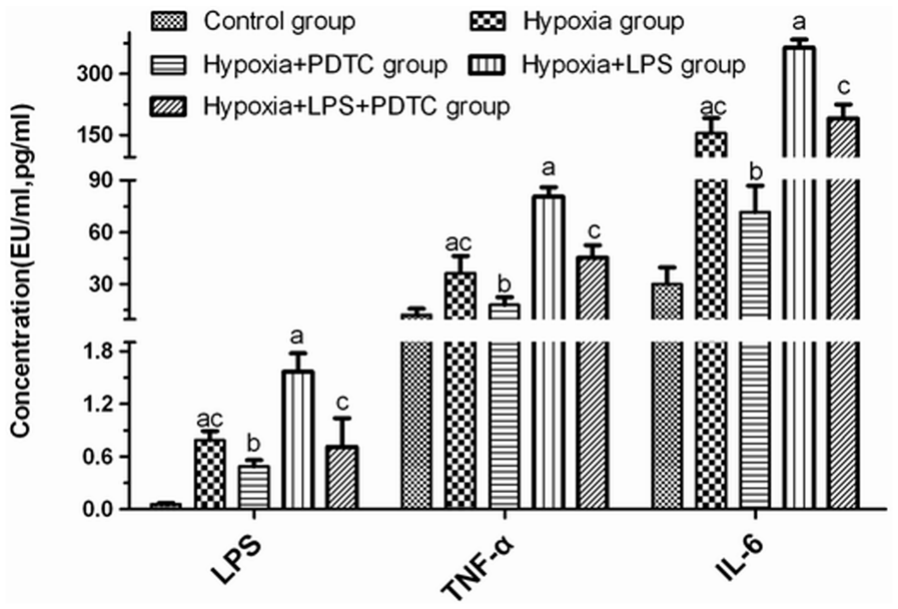
Serum concentration of LPS, TNF-α, and IL-6. Note: ^a^ *P*<0.01 compared to group C; ^b^ *P*<0.01 compared to group H; ^c^ *P*<0.01 compared to group HL.

The serum concentrations of LPS, TNF-α, and IL-6 in groups H and HL were significantly higher than those in group C, suggesting LPS and hypoxia induced inflammatory response. They were significantly higher in group HL (over 2 fold) than in group H (*P*<0.01). The concentrations of LPS, TNF-α, and IL-6 in the serum of rats from group HP were significantly lower than the concentrations in those from group H, and those in group HPL were significantly lower than in group HL (*P*<0.01; **Figure 1**). This result suggested that PDTC-treatment reversed hypoxia and LPS-induced increase of inflammatory mediators.

### Observation of ultrastructure of the intestinal tract using TEM

**Figure 2:**
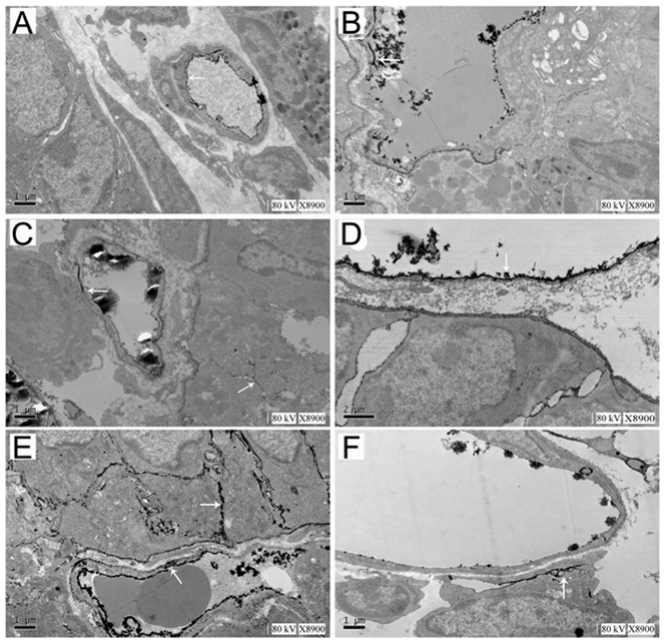
Changes in the ultrastructure of rat intestinal capillaries. **A** In the control group, lanthanum particles were only distributed on capillary lumens; no La^3+^ sediment was observed on the basement membrane lateral or extravascular mesenchyme. The morphology of nuclei and organelles such as mitochondria and the Golgi complex was normal. TEM, ×8900. **B** In the hypoxia group, La^3+^ particles were distributed along the vessel basement membrane. Mild swelling of the organelles was observed. TEM, ×8900. **C** In the hypoxia group, many La^3+^ particles were found to penetrate the basement membrane through the vessel TJs into tissue space, and La^3+^ sediments were observed in the tissues of surrounding vessel. TEM, ×8900. **D** In the HP group, La^3+^ particles were limited to capillary lumens. No see La^3+^ sediment on the basement membrane lateral or surrounding mesenchyme. No notable abnormalities of organelles were found. TEM, ×8900. **E** In the HL group, La^3+^ particles were distributed diffusively in the vessel the inside of basement membrane, A large number of La^3+^ particles penetrate the basement membrane into the TJs, the surrounding vessel endothelial TJs space were enlarged. TEM, ×8900. **F** In the HPL group, La^3+^ particles were distributed in vessel lumens, and only in the local endothelial basement membrane. No La^3+^ particles were observed in the tissue space of surrounding vessel. TEM, ×8900.

In group C, high-density La^3+^ particles were only found attached to the surfaces of small intestinal wall capillary lumens (**Figure 2A**). In group H, La^3+^ particles linearly distributed in vascular endothelial tight junctions (**Figure 2B**, white arrow), and locally small amounts of La^3+^ particles into the surrounding space by tight junctions (**Figure 2C**, white arrow). In group HP, La^3+^ particles were localized on the surfaces of the vascular endothelial cells. No La^3+^ sediment was found in the basement membrane or extravascular mesenchyme (**Figure 2D**). In group HL, La^3+^ particles were distributed diffusely in the capillary endothelium, basement membrane, and interstitial space (**Figure 2E**, white arrows). The sub-epithelial tight junctions (TJs) were open, a large number of La^3+^ particles were also found into the cytoplasm. In group HPL, a small number of La^3+^ particles were distributed in the capillary lumen. In some regions, a small amount of La^3+^ particles through the basement membrane (**Figure 2F**, white arrow), and no La^3+^ particles were found distribution in the interstitial space or cytoplasm.

**Figure 3:**
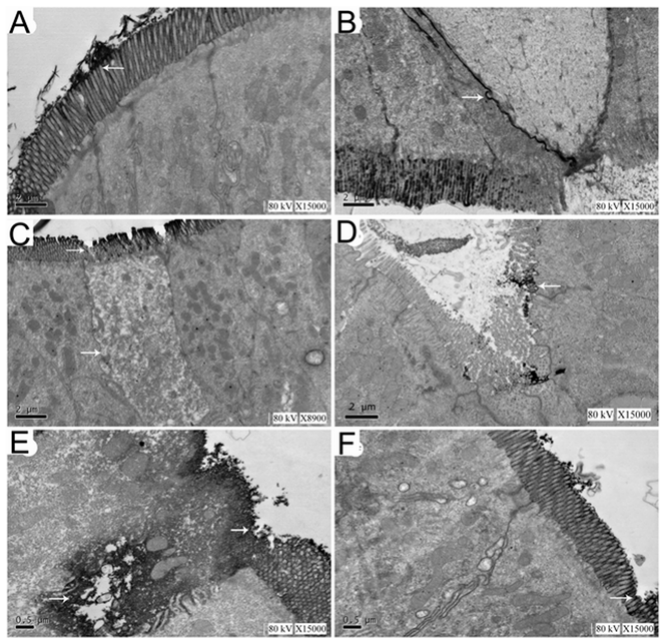
Changes in the ultrastructure of rat intestinal microvilli and interstitial space. **A** In the group C, La^3+^ particles were distributed in the lumen of the small intestine. The number and structure of microvilli were normal. No La^3+^ particles were found in the intercellular space. TEM, ×15,000. **B** In the H group, the TJs between epithelial cells were expanded. La^3+^ particles were observed in the intercellular space and TJs. TEM, ×15,000. **C** In the H group, atrophy and the sloughing of some small intestinal microvilli were observed. Swelling of mitochondria, endoplasmic reticulum, part of the regional organizations is completely dissolved.. TEM, ×8900. **D** In the HP group, La^3+^ particles were observed in the lumen of the intestine. The number of epithelial microvilli was reduced, some microvilli damage,organelle ultrastructure basic normal. No La^3+^ sediment was observed in the intercellular space. TEM, ×15,000. **E** In the HLgroup, Intestinal epithelial cell necrosis, microvilli destruction collapse, organelle dissolution, a large number of La^3+^ particles into the tissue space and cytoplasm. TEM, ×15,000. **F** In the HPL group, microvilli structure is normal, a small amount of of La^3+^ particles attached to the surface of microvilli, and no La^3+^ particles were found in organizational gap and TJs.TEM, ×15,000.

Observation of ultrastructure also showed that in group C, high-density La^3+^ particles were only found attached to the surfaces of small intestinal microvilli (white arrow in **Figure 3A**) No La^3+^ deposition were found in submucosal tissue space. The small intestinal villi were arranged in neat rows and the morphology of the mitochondria, endoplasmic reticulum, and nuclei was normal (**Figure 3A**). In group H, atrophy and sloughing of some small intestinal microvilli were observed, the cytoplasm of some cells was reduced, and transparency was increased. La^3+^ particles were distributed in lines in the junction complexes of the intestinal mucosal epithelium (**Figure 3B**, white arrow). The microvilli of intestinal epithelial atrophied and fell off (**Figure 3C**, white arrows). In group HP, the number of intestinal epithelial microvilli was reduced and showed mild atrophy. La^3+^ particles were localized on the surfaces of the intestinal epithelium and no La^3+^ was found in subepithelial area (**Figure 3D**). In group HL, a large number of intestinal epithelia microvilli shranked or fell off, and cell breakdown and death were observed. La^3+^ particles were distributed diffusely in the intestinal epithelium and interstitial space (**Figure 3E**). In group HPL, a small number of epithelial microvilli sloughed off (white arrow in **Figure 3F**). La^3+^ particles were limited in the intestinal cavity surface. In some regions, La^3+^ particles were found in the capillary basement membrane, and only a small amount of La^3+^ particles were found in the interstitial submucosal space or cytoplasm (**Figure 3F**).

### Detection of TLR4 mRNA levels using real-time fluorescent quantitative RT-PCR

**Figure 4:**
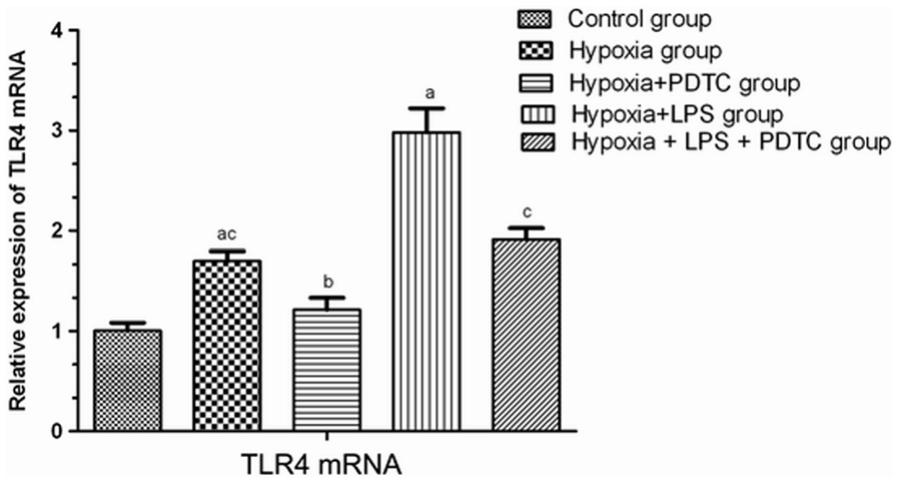
Relative TLR4 mRNA levels in the jejunal tissues of rats from the different groups (n = 7, X¯ ± s). Note: ^a^ *P*<0.01 compared to group C; ^b^ *P*<0.01 compared to group H; ^c^ *P*<0.01 compared to group HL.

The mRNA level of TLR4 from each group was presented in **Figure 4**. Compared to the group C, the TLR4 mRNA levels in the jejunal tissues of groups H and HL were significantly increased (*P*<0.01). The increase in TLR4 expression in group HL was more substantial than that in group H. This suggested that hypoxia and LPS co-treatment dramatically increased TLR4 expression. The TLR4 expression level of group HP was significantly lower than in group H, and the TLR4 expression level of group HPL was significantly lower than in group HL (*P*<0.01). Similarly, inhibiting NF-κB also reversed TLR4 upregulation in response to hypoxia and LPS.

### NF-κB p65 and occludin expression in jejunal tissues

**Figure 5:**
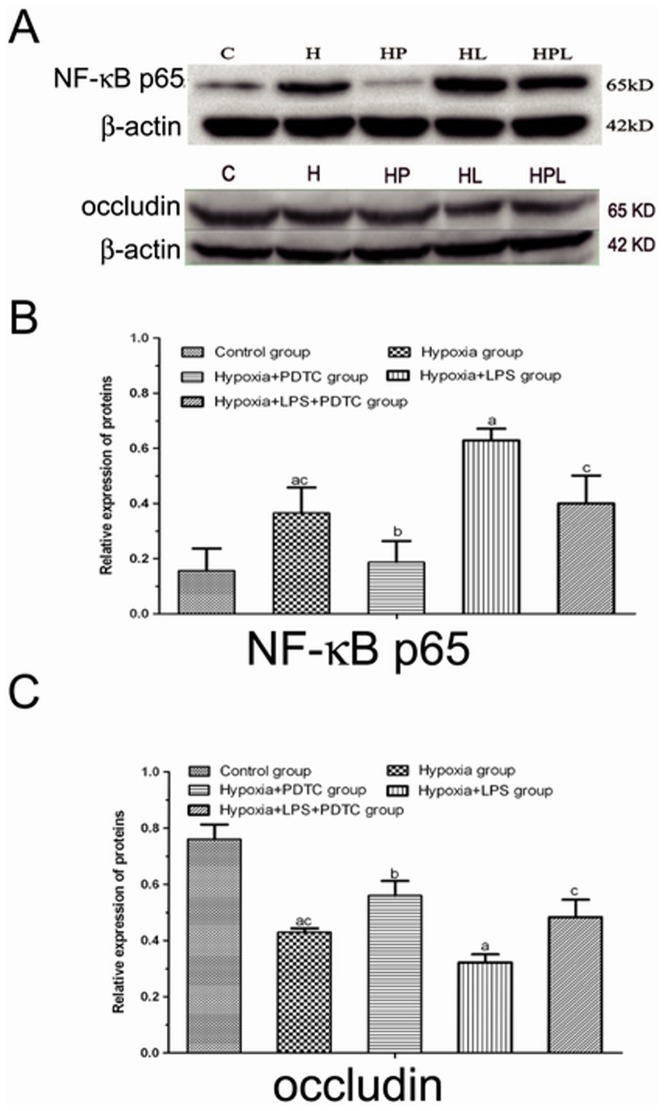
WB detection of protein expressions of NF-κB p65 and occluding. **A** WB result for NF-κB p65 and occludin. **B** Quantification for expression of NF-κB p65 protein. **C** Quantification for expression of occludin protein. ^a^ *P*<0.01 compared to group C; ^b^ *P*<0.01 compared to group H; ^c^ *P*<0.01 compared to group HL.

Western blot (**Figure 5A**) analysis showed that the expression levels of NF-κB p65 in group H (0.366±0.092) and HL (0.629±0.042) were higher than that in group C (0.156±0.080), and this increase was more substantial for group HL than for group H (**Figure 5B**). After PDTC intervention, the expression levels of NF-κB p65 in groups HP (0.187±0.097) and HPL (0.400±0.101) were significantly lower than in groups H and HL, respectively (*P*<0.01). Alternations of occludin protein showed an opposite trend of NF-κB p65 expression (**Figure 5C**). Group H and HL showed significantly decreased expression of occludin compared to the control group. PDTC administration rescued hypoxia and LPS induced reduction of occludin, as shown by increased occludin protein in group HP and HPL.

## Discussion

Special geological and climatic environments at high altitudes might lead to decreased body resistance that increases susceptibility to intestinal diseases in populations recently exposed to high altitudes. In this study, rats exposed to acute hypoxia at a simulated elevation of 7000 m for 3 days showed detectable intestinal bacteria in many organs outside the intestinal tract, suggesting that hypoxia facilitated intestinal bacterial translocation. This is consistent with previous reports of hypoxia-induced bacterial translocation [10]. As functional damage of the intestinal barrier was reflected by changes of the permeability of the intestinal mucosa to large molecules, the rats in group HL were received abdominal injections of low-dose LPS to simulate an exogenous infection. In this way, in addition to bacterial translocation, endotoxin translocation in the intestinal tract was also measured to evaluate damages to the function of the intestinal barrier. Serum analysis showed that serum LPS levels in groups H and HL were significantly higher than in group C. TEM observation of the jejunal mucosa of rats from groups H and HL showed that rat jejunal mucosal microvilli shrank and fell off. The intercellular space between epithelial cells and capillary endothelial cells increased. Lanthanum nitrate particles could reach regions that were not accessible through the intercellular space under normal conditions. These results suggest that exposure to hypoxia causes substantial damage to the function of the rat intestinal barrier and bacterial translocation occurs by some means.

TLR4 plays an important role in the innate intestinal immune system as the first part of the intestinal tract recognizing bacteria. It is not only an immune recognition receptor on the cell surface but also a transmembrane signal transduction molecule. During the development of enteric septicemia, LPS, a strong activator of TLR4 in the intestinal epithelium, specifically binds to TLR4. TLR4 then activates the MyD88-dependent and TRIF-dependent pathways and thereby activates intracellular signaling molecules such as the IRAKs, TRAFs, and TAK1. Thus, it ultimately forms the primary and secondary signal waves that activate NF-κB. NF-κB induces the transcription and translation of inflammatory cytokines and leads to the massive release of inflammatory mediators [11]–[13]. In local regions, these molecules can lead to apoptosis of intestinal mucosal epithelial cells and damage the tissues and organs of the intestinal tract. It can also act on distant organs and amplify systemic inflammatory reactions. Currently, studies on the role of TLR4 in intestinal immune function have not generated consistent results. Fukata et al. found that TLR4 could promote the proliferation of epithelial cells and inhibit intestinal bacterial translocation [14]. However other studies showed that, compared to wild-type mice, intestinal epithelial damage caused by colitis was milder in TLR4-deficient mice [15], [16]. The infiltration of cytokines, macrophages, and neutrophils was reduced. These results suggest that TLR4 has different functions in different cellular events. The functions and mechanisms of TLR4 and NF-κB with respect to damage to the function of the intestinal barrier and to bacterial translocation under hypoxic conditions have not yet been reported. In this study, real-time fluorescent quantitative RT-PCR and western blot experiments showed that, in group H, the expression of TLR4 and NF-κB was elevated in jejunal tissues, and these elevations were more substantial when LPS was added. These results suggest that hypoxia alone can upregulate TLR4 expression and activate the TLR4/NF-κB signaling pathway; hypoxia and infection can further aggravate such phenomena. TNF-α and IL-6 are important inflammatory factors located downstream from NF-κB. They play important roles in various inflammatory reactions and are highly correlated with the severity of inflammation [17]. In our study, by detecting TNF-α and IL-6, the degree of activation of this signaling pathway was found the TLR4/NF-κB to be consistent with changes in the rate of intestinal bacterial translocation, serum levels of endotoxin, and damage to the ultrastructure of the intestinal mucosa. Soares et al. proposed that TLR4 might play an important role in recruiting granulocytes after intestinal damage and in the inflammatory reaction caused by bacterial translocation [18]. Therefore, we deduced that damage to the function of the intestinal barrier and bacterial translocation under hypoxic conditions might be closely related to the TLR4/NF-κB signaling pathway.

PDTC is a specific inhibitor of NF-κB. It inhibits the nuclear translocation of the NF-κB p65 subunit by reducing IκB degradation; thus, it inhibits the expression of downstream cytokines. The current study also found that PDTC could significantly reduce TLR4 mRNA levels, possibly via positive feedback after blockage of NF-κB or via some direct inhibitory effect of PDTC on TLR4 expression. However, the exact mechanism merits further study. Fluorescent quantitative RT-PCR and western blot analysis showed that, after PDTC administration, the expression levels of TLR4 and NF-κB in groups HP and HPL were significantly downregulated, accompanied by decreases in the intestinal bacterial translocation rate and serum levels of endotoxin and inflammatory cytokines. TEM showed that the ultrastructures were mostly normal. These results further demonstrate that the TLR4/NF-κB signaling pathway is involved in damage to the function of the intestinal barrier and in bacterial translocation under hypoxic conditions.

TLR4 and MD2 form symmetrical (M)-shaped polymers, which are widely distributed on the surface of intestinal cell membranes. After LPS binds to LBP and CD14, which are anchored to the membrane, the TLR4-MD2 complex is activated and intracellular signal transfer begins [19]. Studies have found that they are also expressed on the surface of intestinal epithelial cells and antigen-presenting cells such as dendritic cells and macrophages. In this study, simulated plateau low-pressure hypoxia caused not only disturbance of the intestinal microecology, flora imbalance, and massive proliferation of dominant bacteria (gram-negative *Escherichia coli*) but also the accumulation of harmful substances in the intestinal tract. Changes in the localization of intestinal bacteria and disturbance of the dynamic balance of their numbers for any reason can cause dominant bacteria to break through the mucosa and cause bacterial translocation, inducing intestinal infections [20], [21]. For example, intestinal endotoxemia caused by bacterial translocation can aggravate damage to the intestinal mucosa and further accelerate bacterial translocation [22]. The insufficient energy synthesis caused by hypoxia, however, decreases the frequency of cilia swing, slows peristalsis, and inhibits self-cleaning in the intestinal tract. In addition, the blood and oxygen supplies required for the normal functioning of the intestinal mucosa vary greatly under different conditions. The special anatomical structures of the intestinal microvilli were extremely sensitive to hypoxia, Under physiological conditions, the epithelial microvilli are already in a hypoxic state, and this is aggravated under inflammatory conditions [23], [24]. External low-pressure hypoxic environments can also aggravate damage to the microvilli [25]. Many different factors can give intestinal bacteria more chances to contact intestinal mucosal epithelial cells and various antigen-presenting cells, and adhesion time increases. After the LPS interact with the TLR4 complexes on the cell surface, intracellular signal molecule cascades become activated. With the participation of multiple intracellular proteins, NF-κB is ultimately activated and translocates to the nucleus, where it induces the pronounced expression of inflammation-related genes. These massive amounts of inflammatory molecules are released to mediate the intestinal inflammatory reaction. In this experiment, TNF-α and IL-6 serum levels were measured. The results showed that the changes in cytokine content were consistent with the changes in the bacterial translocation rate and serum levels of LPS. The TNF-α and IL-6 levels in groups H and HL were both significantly increased. After PDTC intervention, the downregulation of TLR4 and the blocking of the TLR4/NF-κB signaling pathway significantly decreased the levels of 3 indicator cytokines, TLR4, and NF-κB, suggesting that, under hypoxic conditions, bacterial LPS bound to TLR4 ultimately activates NF-κB, thus mediating the inflammatory response and damaging the function of the intestinal barrier. TNF-α is an important monokine. It is located downstream from NF-κB and is involved in the occurrence and development of most inflammatory reactions. Studies have shown that the function of the intestinal barrier may be regulated by a network of multiple cytokines, including TNF-α, INFs, and ILs [26]. TNF-α can cause apoptosis of intestinal epithelial cells. In addition, Cui et al. found that TNF-α can lead to decreased phosphorylation of claudin-1, a major protein for closure of TJ complexes, and dissociate claudin-1 from TJs [27]. Thus, TJs are broken and mucosal permeability increases. More studies have demonstrated that TNF-α can change the expression and localization of occludin and ZO-1, increasing the permeability of the intestinal mucosa, which promotes bacterial translocation. All of these changes contribute to the occurrence of MODS [28]. IL-6 is another important inflammatory mediator induced by NF-κB activation. It plays an important role in the inflammatory reactions of intestinal and distant organs under hypoxic conditions. It was once thought that IL-6 is secreted mainly by epithelial and immune cells; however, Walton et al. found that subepithelial myofibroblasts could also secrete large amounts of IL-6 through TLR4/NFκB-mediated pathways, which then participated in the intestinal inflammatory reaction. Takuya et al. found that IL-6 could induce the upregulation of claudin-2, a channel protein that contributes to the formation of TJ complexes, thus increasing mucosal permeability [29]. This suggests that after the TLR4/NF-κB signaling pathway is activated under hypoxic conditions, the transcription and translation of downstream inflammatory mediators increases. These inflammatory mediators are released into the blood and act on local intestinal mucosal epithelial cells as a feedback mechanism, resulting in a secondary attack, which further aggravates bacterial translocation by damaging the structure of TJs and aggravating the impairment of the function of the intestinal barrier. In this way, a destructive cycle is formed. In addition to the aforementioned secondary effects, TLR4/NF-κB may participate directly in epithelial barrier damage and bacterial translocation. Thomas et al. reported that invasive bacteria could lead to changes in TJ protein expression via TLR-mediated pathways [30]. A similar downregulation of claudin could be caused by different bacterial strains. Further *in vivo* experiments showed that LPS-induced TLR4 upregulation could inhibit the expression of claudin-7, damaging the barrier and promoting bacterial translocation. There is a great deal of TLR4 expression in the intestinal tract, and the discovery of its expression provided a new point of view in the study of intestinal bacterial translocation under hypoxic conditions. This fosters another means of explaining the mechanism underlying what we observed in the current study, i.e., the aggravated damage to the function of the intestinal barrier and increased bacterial translocation were caused by the upregulation of TLR4 after activation by LPS.

The previous studies about the impact of TLR4 on TJ mechanisms has been focused on the claudin protein family and its subtypes, while the involvement of occludin in the formation of intestinal TJ complexes under hypoxia has been rarely reported. Occludin is a separate TJ transmembrane protein identified by Furuse et al. [31]; it forms the outer ring tight gap between the “zipper” structure blocked cells and is directly involved in the formation of the TJ. The absence of occludin increases the ion permeability of TJs [32] suggested an important role of occludin in the maintenance of TJ functions. In this study we observed that hypoxia decreased TLR4/NF-κB expression while increased occludin expression. The expression of TLR4/NF-κB signaling proteins were inversely correlated with the level of occludin in response to hypoxia and hypoxia with PDTC, suggesting that the activation of the TLR4/NF-κB may have a close relationship with the change in occludin expression under hypoxic condition. Peterson et al. [33] showed that thermal damage to intestinal permeability increased distant organ injury that was associated with significantly reduced occludin expression and TLR4 activation, while this injury was attenuated in TLR4-deficient mice. Sheth et al. [34] reported that LPS-induced TLR4 activation in biliary epithelial tissue increased the phosphorylation of threonine/serine residues of occludin and thus destroyed the epithelial barrier. In addition, activation of TLR4 can also alter the cellular localization of occludin. De La Serre et al. [35] reported an upregulation of intestinal TLR4 associated with redistribution of cellular occludin from cell membrane to intracellular area in obese SD rats fed a high-fat diet. This redistribution of occludin might damage barrier function. Thus, hypoxia-induced activation of TLR4/NF-κB may influence TJ complexes through affecting occuldin expression and distribution that eventually lead to the dysfunction of the paracellular pathway, cause damage to the intestinal barrier, and result in bacterial translocation.

The paracellular and transcellular pathways are two typical modes for the transmembrane transfer of intestinal bacteria and substances. Thomas et al. pointed out that the current view that bacteria are usually transferred via a mechanical, physical pathway may not be completely correct [30]. *In vivo* and *in vitro* experiments by Matthew et al. showed that *E. coli* could enter the cells via endocytosis by forming phagosomes [36] and this process was mediated by upregulation of TLR4 in the non-immune intestinal cells, epithelium,. LPS was also found to upregulate TLR4 expression and promote endocytosis. The bacteria survived inside the cells after endocytosis and were translocated in pinocytic vesicles, which can occur even when the mechanical intestinal epithelial barrier is intact. Depletion or blocking with antagonist of TLR4 effectively inhibits bacterial endocytosis and metastasis, suggesting that aside from paracellular pathways, the extra-intestinal transfer of bacteria can also occur via transcellular pathways mediated by TLR4. In the current study, the observation that PDTC inhibited hypoxia-induced TLR4 expression and increase of bacterial translocation further supports viewpoint expressed above.

In summary, we found that activation of TLR4/NF-κB signaling pathway contributes to hypoxia-caused damage to the function of the intestinal barrier and bacterial translocation.. Plateau low-pressure hypoxia promotes changes in the morphology and function of intestinal microvilli and flora imbalance in the intestine. Bacterial LPS then interacts with epithelial TLR4 and activates the TLR4/NF-κB signaling pathway. Activated NF-κB is translocated into the nucleus, where it induces the transcription and translation of downstream inflammatory factors, thereby causing a massive release of TNF-α and IL-6 into the blood which mediates inflammatory reaction and act on distant organs or even the whole body. Inhibiting this pathway can effectively alleviate those damages. In addition, the decreased expression of occludin, an important component of intestinal TJ complexes, may aggravate the damage and promotes bacterial translocation. However, whether endocytosis-mediated transcellular transfer of bacteria occurs simultaneously with LPS and TLR4/NFκB mediated damage of TJ structure during hypxia-induced damage of intestinal barrier and bacterial translocation merits further investigation.

The incidence of gastrointestinal tract diseases is high in high-altitude environments, because the gastrointestinal tract is usually the place where MODS begins. Barrier damage-induced intestinal infections are often involved in the development of many severe high-altitude diseases. Understanding the TLR4/NF-κB signaling pathway may provide a new target for the prevention of severe high-altitude diseases.

## References

[1] Zhou B, Yang DZ, Zhou QQ (2009) The SEM observation of small intestina lmucosa in the rabbits under simulated high altitude hypoxia. Chin J Ggastroenterol Hepatol 18: 751–753.

[2] Recavarren-Arce S, Ramirez-Ramos A, Gilman RH, Chinga-Alayo E, Watanabe-Yamamoto J, et al (2005) Severe gastritis in the Peruvian Andes. Histopathology 46: 374–379. 15810948 10.1111/j.1365-2559.2005.02102.x

[3] Deitch EA (2002) Bacterial translocation or lymphatic drainage of toxic products from the gut: what is important in human beings? Surgery 131: 241–244. 11894026 10.1067/msy.2002.116408

[4] Wang ZT, Yao YM, Xiao GX, Sheng ZY (2004) Risk factors of development of gut-derived bacterial translocation in thermally injured rats. World J Gastroenterol 10: 1619–1624. 15162536 10.3748/wjg.v10.i11.1619 PMC4572765

[5] Walser EM, Nealon WH, Marroquin S, Raza S, Hernandez JA, et al (2006) Sterile fluid collections in acute pancreatitis: catheter drainage versus simple aspiration. Cardiovasc Intervent Radiol 29: 102–107. 16283578 10.1007/s00270-004-0220-4

[6] Spehlmann ME, Eckmann L (2009) Nuclear factor-kappa B in intestinal protection and destruction. Curr Opin Gastroenterol 25: 92–99. 19528876 10.1097/MOG.0b013e328324f857

[7] Zhang FX, Yang WC, Deng ZY, Wu WM, Wu HP, et al (2010) Effect of glutamine on change of intestinal microecology in rats exposed to acute high altitude. Chinese Journal of Microecology 22: 1–4.

[8] Igawa S, Kishibe M, Murakami M, Honma M, Takahashi H, et al (2011) Tight junctions in the stratum corneum explain spatial differences in corneodesmosome degradation. Exp Dermatol 20: 53–57. 20955201 10.1111/j.1600-0625.2010.01170.x

[9] Wei L, Yin Z, Yuan Y, Hwang A, Lee A, et al (2010) A PKC-beta inhibitor treatment reverses cardiac microvascular barrier dysfunction in diabetic rats. Microvasc Res 80: 158–165. 20079359 10.1016/j.mvr.2010.01.003

[10] Zhou QQ, Yang DZ, Luo YJ, Liu FY (2011) Over Starvation Aggravate Intestinal Mucosal Injury and Promote Bacterial Translocation Under High-Altitude Hypoxic Environment Exposure. World Gastroenterology 17: 1584–1593. 10.3748/wjg.v17.i12.1584 PMC3070130 21472125

[11] Noreen M, Shah MA, Mall SM, Choudhary S, Hussain T, et al (2012) TLR4 polymorphisms and disease susceptibility. Inflamm Res 61: 177–188. 22277994 10.1007/s00011-011-0427-1

[12] Wullaert A (2010) Role of NF-kappaB activation in intestinal immune homeostasis. Int J Med Microbiol 300: 49–56. 19781989 10.1016/j.ijmm.2009.08.007

[13] Covert MW, Leung TH, Gaston JE, Baltimore D (2005) Achieving stability of lipopolysaccharide-induced NF-kappaB activation. Science 309: 1854–1857. 16166516 10.1126/science.1112304

[14] Fukata M, Michelsen KS, Eri R, Thomas LS, Hu B, et al (2005) Toll-like receptor-4 is required for intestinal response to epithelial injury and limiting bacterial translocation in a murine model of acute colitis. Am J Physiol Gastrointest Liver Physiol 288: G1055–G1065. 15826931 10.1152/ajpgi.00328.2004

[15] Heimesaat MM, Fischer A, Siegmund B, Kupz A, Niebergall J, et al (2007) Shift towards pro-inflammatory intestinal bacteria aggravates acute murine colitis via Toll-like receptors 2 and 4. PLoS One 2: e662. 17653282 10.1371/journal.pone.0000662 PMC1914380

[16] Ohkawara T, Takeda H, Nishihira J, Miyashita K, Nihiwaki M, et al (2005) Macrophage migration inhibitory factor contributes to the development of acute dextran sulphate sodium-induced colitis in Toll-like receptor 4 knockout mice. Clin Exp Immunol 141: 412–421. 16045730 10.1111/j.1365-2249.2005.02877.x PMC1809451

[17] Van Deventer SJ (1997) Tumour necrosis factor and Crohn's disease. Gut 40: 443–448. 9176068 10.1136/gut.40.4.443 PMC1027115

[18] Soares AL, Coelho FR, Guabiraba R, Kamal M, Vargaftig BB, et al (2010) Tumor necrosis factor is not associated with intestinal ischemia/reperfusion-induced lung inflammation. Shock 34: 306–313. 20160673 10.1097/SHK.0b013e3181cdc585

[19] Buchholz BM, Bauer AJ (2010) Membrane TLR signaling mechanisms in the gastrointestinal tract during sepsis. Neurogastroenterol Motil 22: 232–245. 20377787 10.1111/j.1365-2982.2009.01464.x PMC2951022

[20] Shimizu K, Ogura H, Goto M, Asahara T, Nomoto K, et al (2006) Altered gut flora and environment in patients with severe SIRS. J Trauma 60: 126–133. 16456446 10.1097/01.ta.0000197374.99755.fe

[21] Almeida J, Galhenage S, Yu J, Kurtovic J, Riordan SM (2006) Gut flora and bacterial translocation in chronic liver disease. World J Gastroenterol 12: 1493–1502. 16570339 10.3748/wjg.v12.i10.1493 PMC4124279

[22] Bellot P, Frances R, Such J (2008) Bacterial translocation in cirrhosis. Gastroenterol Hepatol 31: 508–514. 18928751 10.1157/13127094

[23] Gao JS, Yang SL (2009) Advance in causes and mechanisms of intestinal injury. World Chinese Journal of Digestology 17: 1540–1544.

[24] Kelly CJ, Colgan SP (2012) Targeting Hypoxia to Augment Mucosal Barrier Function. J Epithel Biol Pharmacol 5: 67–76. 28824735 10.2174/1875044301205010067 PMC5560425

[25] Wu WM, Zhang FX, Zhang P (2010) Influence of plateau hypoxia on the tissue injury and expression of HIF-1α and iNOS in intestinal mucosa of rats. Med J Chin PLA 35: 592–594.

[26] Xavier RJ, Podolsky DK (2007) Unravelling the pathogenesis of inflammatory bowel disease. Nature 448: 427–434. 17653185 10.1038/nature06005

[27] Cui W, Liu DY, Ma L, Liu P (2007) Effect of tumor necrosis factor-α on protein expression of tight junction protein in intestinal epithelial cells. World Chinese Journal of Digestology 15: 1788–1793.

[28] Farrell CP, Barr M, Mullin JM, Lande L, Zitin M (2012) Epithelial Barrier Leak in Gastrointestinal Disease and Multiorgan Failure. J Epithel Biol Pharmacol 5: 13–18.

[29] Suzuki T, Yoshinaga N, Tanabe S (2011) Interleukin-6 (IL-6) regulates claudin-2 expression and tight junction permeability in intestinal epithelium. J Biol Chem 286: 31263–31271. 21771795 10.1074/jbc.M111.238147 PMC3173073

[30] Clarke TB, Francella N, Huegel A, Weiser JN (2011) Invasive bacterial pathogens exploit TLR-mediated downregulation of tight junction components to facilitate translocation across the epithelium. Cell Host Microbe 9: 404–414. 21575911 10.1016/j.chom.2011.04.012 PMC4536975

[31] Furuse M, Hirase T, Itoh M, Nagafuchi A, Yonemura S, et al (1993) Occludin: a novel integral membrane protein localizing at tight junctions. J Cell Biol 123: 1777–1788. 8276896 10.1083/jcb.123.6.1777 PMC2290891

[32] Yu AS, Mccarthy KM, Francis SA, McCormack JM, Lai J, et al (2005) Knockdown of occludin expression leads to diverse phenotypic alterations in epithelial cells. Am J Physiol Cell Physiol 288: C1231–C1241. 15689410 10.1152/ajpcell.00581.2004

[33] Peterson CY, Costantini TW, Loomis WH, Putnam JG, Wolf P, et al (2010) Toll-like receptor-4 mediates intestinal barrier breakdown after thermal injury. Surg Infect (Larchmt) 11: 137–144. 20374005 10.1089/sur.2009.053 PMC3304242

[34] Sheth P, Delos SN, Seth A, LaRusso NF, Rao RK (2007) Lipopolysaccharide disrupts tight junctions in cholangiocyte monolayers by a c-Src-, TLR4-, and LBP-dependent mechanism. Am J Physiol Gastrointest Liver Physiol 293: G308–G318. 17446308 10.1152/ajpgi.00582.2006

[35] De La Serre CB, Ellis CL, Lee J, Hartman AL, Rutledge JC, et al (2010) Propensity to high-fat diet-induced obesity in rats is associated with changes in the gut microbiota and gut inflammation. Am J Physiol Gastrointest Liver Physiol 299: G440–G448. 20508158 10.1152/ajpgi.00098.2010 PMC2928532

[36] Neal MD, Leaphart C, Levy R, Prince J, Billiar TR, et al (2006) Enterocyte TLR4 mediates phagocytosis and translocation of bacteria across the intestinal barrier. J Immunol 176: 3070–3079. 16493066 10.4049/jimmunol.176.5.3070

